# Duodenal stricture in Crohn’s disease successfully managed with a fully covered metal stent-assisted with double pig-tail stents

**DOI:** 10.1055/a-2589-0790

**Published:** 2025-05-09

**Authors:** Changqing Sun, Juan Wei, Yuping Qiu, Shupei Li, Juan Xu, Xiaoli Ren, Ji Xuan

**Affiliations:** 1 12461 Department of Gastroenterology, Jinling Clinical Medical College, Nanjing Medical University, Nanjing, China; 2 144990 Department of Gastroenterology, Jinling Hospital, Affiliated Hospital of Medical School, Nanjing University, Nanjing, China; 3 144990 Department of Gastroenterology, Jinling Clinical Medical College, Nanjing University of Chinese Medicine, Nanjing, China

A fully covered metal stent is a potentially feasible treatment option for refractory benign strictures, though stent migration limits its use 1 2 .

We present the case of a 21-year-old man diagnosed with Crohn’s disease (A2L3+4B2, CDAI: 59.14), who presented with a long-segment pyloroduodenal obstruction secondary to upper gastrointestinal Crohn’s disease. The patient presented with abdominal pain, vomiting, and significant weight loss, resulting in malnutrition. Endoscopy revealed pyloroduodenal stricture ( Fig. 1 ). Upper gastrointestinal radiography showed a delayed passage of contrast medium through the pyloroduodenal region ( Fig. 2 ). The patient received a 390 mg intravenous dose of ustekinumab. However, the obstruction persisted, and conservative anti-inflammatory treatment proved ineffective.

**Fig. 1:**
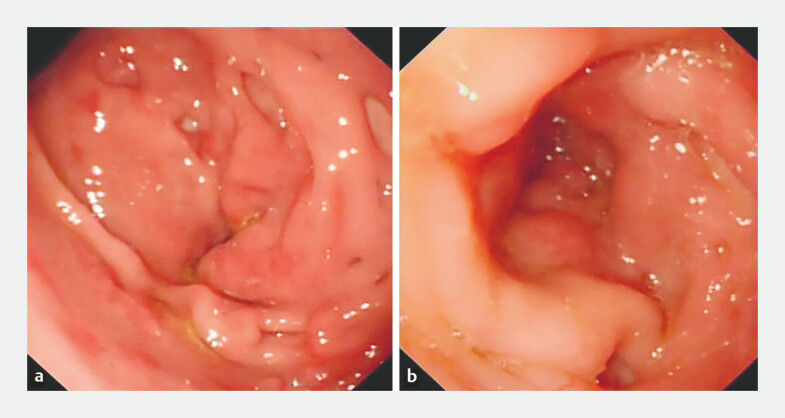
Endoscopy revealed pyloroduodenal stricture.

**Fig. 2:**
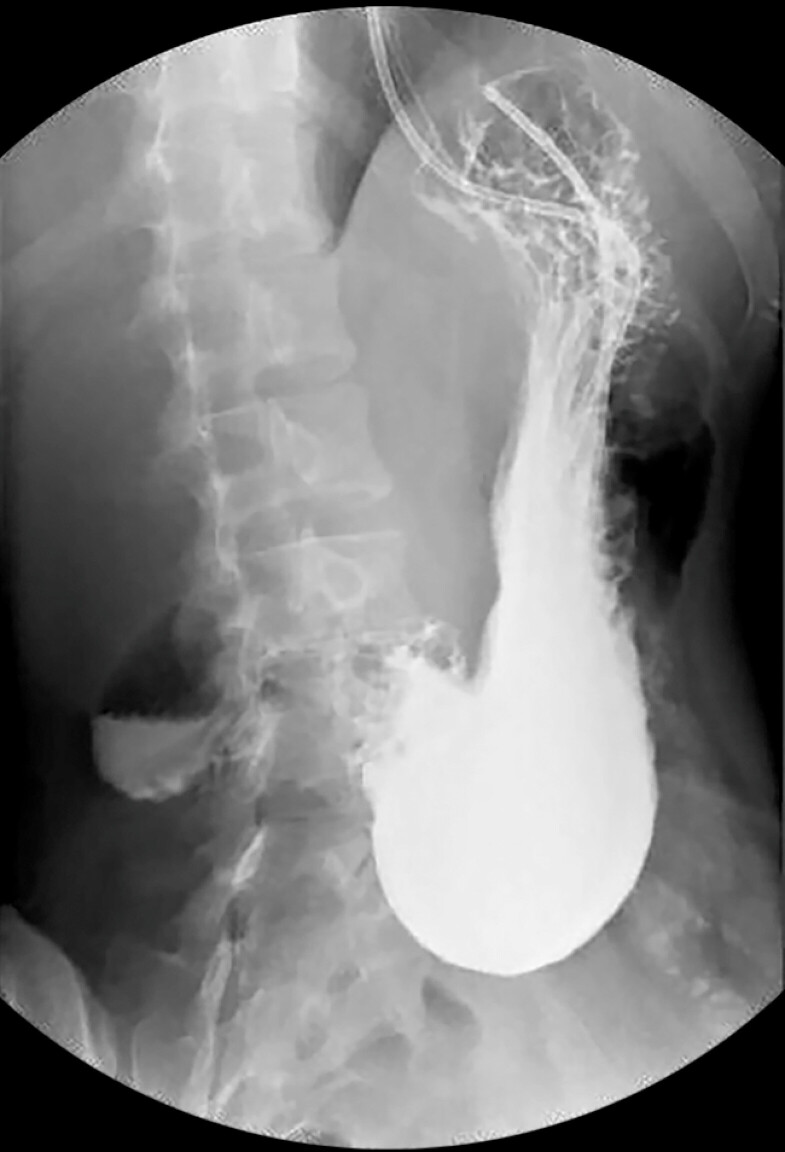
Upper gastrointestinal radiography showed a delayed passage of contrast medium through the pyloroduodenal region.

The patient declined surgery. The stricture is located in an anatomically curved and sharply angulated region. Endoscopic balloon dilation is suitable for short (<5 cm long) and straight strictures 3 ; however, in this case, the stricture was curved, and its length >5 cm. Consequently, we opted to place a fully covered metal stent.

A fully covered stent was deployed at the site of stricture. Subsequently, two double pig-tail plastic stents were inserted in a perpendicular configuration to form an “X” effectively fixing the fully covered stent. The patient had membrane damage by local granulation tissue infiltration into the stent 2 weeks after stent placement. To prevent mucosal injury, the stent was removed using an inverted technique via the anal route successfully ( Video 1 ). Postoperatively, the patient received symptomatic treatment, including acid suppression, gastric mucosal protection, and regular ustekinumab therapy for the underlying disease. Three months later, no evidence of recurrent strictures was observed.

Two double pig-tail stents in a perpendicular configuration, successfully fixed the fully covered stent.Video 1

This novel technique aims to reduce stent migration. Fully covered duodenal stent placement holds promise as a safe and effective treatment for refractory benign gastrointestinal strictures, offering the potential to delay or obviate the need for surgery.

Endoscopy_UCTN_Code_TTT_1AO_2AZ

**Correction**

**Correction: Duodenal stricture in Crohn’s disease successfully managed with a fully covered metal stent-assisted with double pig-tail stents** Changqing Sun, Juan Wei, Yuping Qiu et al. Duodenal stricture in Crohn’s disease successfully managed with a fully covered metal stent-assisted with double pig-tail stents. Endoscopy 2025; 57: E394–E395, doi:10.1055/a-2589-0790 In the above-mentioned article affiliation 1 has been corrected. This was corrected in the online version on March 13, 2026.

## Notes

Endoscopy E-Videos https://eref.thieme.de/e-videos
*E-Videos* is an open access online section of the journal *Endoscopy* , reporting on interesting cases and new techniques in gastroenterological endoscopy. All papers include a high-quality video and are published with a Creative Commons CC-BY license. Endoscopy E-Videos qualify for HINARI discounts and waivers and eligibility is automatically checked during the submission process. We grant 100% waivers to articles whose corresponding authors are based in Group A countries and 50% waivers to those who are based in Group B countries as classified by Research4Life (see: https://www.research4life.org/access/eligibility/ ). This section has its own submission website at https://mc.manuscriptcentral.com/e-videos .

## Notes

Correction **Correction: Duodenal stricture in Crohn’s disease successfully managed with a fully covered metal stent-assisted with double pig-tail stents** Changqing Sun, Juan Wei, Yuping Qiu et al. Duodenal stricture in Crohn’s disease successfully managed with a fully covered metal stent-assisted with double pig-tail stents. Endoscopy 2025; 57: E394–E395, doi:10.1055/a-2589-0790 In the above-mentioned article affiliation 1 has been corrected. This was corrected in the online version on March 13, 2026.
