# Correction: Duodenal stricture in Crohn’s disease successfully managed with a fully covered metal stent-assisted with double pig-tail stents

**DOI:** 10.1055/a-2832-8007

**Published:** 2026-03-13

**Authors:** Changqing Sun, Juan Wei, Yuping Qiu, Shupei Li, Juan Xu, Xiaoli Ren, Ji Xuan

**Affiliations:** 1 12461 Department of Gastroenterology, Jinling Clinical Medical College, Nanjing Medical University, Nanjing, China; 2 144990 Department of Gastroenterology, Jinling Hospital, Affiliated Hospital of Medical School, Nanjing University, Nanjing, China; 3 144990 Department of Gastroenterology, Jinling Clinical Medical College, Nanjing University of Chinese Medicine, Nanjing, China



In the above-mentioned article affiliation 1 has been corrected. This was corrected in the online version on March 13, 2026.

